# Artificial Intelligence and Machine Learning in the Diagnosis and Management of Osteoporosis: A Comprehensive Review

**DOI:** 10.3390/medicina62010027

**Published:** 2025-12-23

**Authors:** Alessandro Conforti, Marco Ruggiero, Linda Lucchetti, Valerio Cipolloni, Francesco Demostene Galati, Martina Gentile, Alberto Lo Gullo

**Affiliations:** 1 ASL Roma 4, 00053 Rome, Italy; 2 Spinal Unit, CTO Hospital, ASL Roma 2, 00159 Rome, Italy; 3 Hospital Pharmacy Unit, ASL Roma 4, 00053 Rome, Italy; 4 Department of Orthopaedics, A.O.U. “Vanvitelli” University Hospital, “Luigi Vanvitelli” University, Via del Sole 10, 80138 Naples, Italy; 5 Fondazione Policlinico Universitario Campus Bio Medico, 00128 Rome, Italy; 6 Ambulatorio Reumatologia Montefiascone—Bagnoregio, ASL Viterbo, 01100 Rome, Italy; 7 UOSD Reumatologia, A.O. Papardo, 98158 Messina, Italy

**Keywords:** artificial intelligence, osteoporosis, machine learning

## Abstract

*Background and Objectives*: Osteoporosis is a prevalent skeletal disorder characterized by decreased bone mass and compromised bone microarchitecture, leading to an elevated risk of fractures and significant morbidity, particularly among aging populations. Early diagnosis and personalized management are critical to reducing fracture incidence and associated healthcare burdens. Recent advances in artificial intelligence (AI) and machine learning (ML) have led to potential improvements in enhancing osteoporosis care by enabling accurate diagnostic imaging analysis, robust fracture risk prediction, and personalized therapeutic strategies. *Materials and Methods*: We performed a narrative review to summarize and critically evaluate the current literature on AI and ML applications in osteoporosis diagnosis and management. We searched relevant literature from inception to January 2025 to provide a comprehensive perspective, focusing on key themes, methodological approaches, and clinical implications. *Results*: Deep learning models, especially convolutional neural networks, facilitate rapid and accurate bone mineral density assessment from routine radiographs, expanding screening capabilities beyond conventional dual-energy X-ray absorptiometry (DXA). Machine learning algorithms harness clinical and demographic data to generate fracture risk models that often outperform traditional tools, enabling timely identification of high-risk individuals. Furthermore, AI-driven analyses of historical treatment responses coupled with real-time monitoring through wearable technologies and mobile applications allow for personalized therapeutic optimization and enhance patient engagement. Despite these promising advances, challenges remain regarding ethical considerations, data privacy, legal liability, incomplete model validation, lack of standardization, and the need for critical appraisal of real-world clinical efficacy for widespread clinical adoption. *Conclusions*: This narrative review indicates that AI and ML hold significant promise to revolutionize osteoporosis management by enabling early detection, precise risk stratification, and tailored interventions. However, the current evidence is heterogeneous, often lacking robust external validation and quantitative synthesis. Critical gaps include insufficient evaluation of model robustness across diverse populations, discussion of negative or conflicting results, and a comprehensive assessment of the limitations inherent in current AI evidence. Strategic efforts to validate, regulate, and critically integrate these technologies into routine clinical workflows are essential to realize their full potential and address the growing burden of osteoporosis worldwide.

## 1. Introduction

Osteoporosis is a systemic skeletal disorder characterized by decreased bone mass and microarchitectural deterioration of bone tissue, leading to increased bone fragility and a higher risk of fractures. Clinically, osteoporosis represents a major public health concern due to its high prevalence, particularly among postmenopausal women and the elderly population, and the associated substantial morbidity, mortality, and healthcare costs associated with osteoporotic fractures [1]. Worldwide, osteoporosis causes more than 8.9 million fractures annually, and the direct economic burden is estimated to be tens of billions of dollars each year [1,2]. Fragility fractures, especially those of the hip, vertebrae, and wrist, significantly impair mobility and quality of life, underscoring the critical need for timely diagnosis and effective management of this silent disease [3].

Artificial intelligence (AI) and machine learning (ML) encompass transformative computational technologies that simulate human cognitive processes to analyze complex datasets, discern patterns, and generate predictive models. AI broadly refers to systems capable of performing tasks that typically require human intelligence, such as image recognition, natural language processing, and decision-making [4]. ML, a subset of AI, involves algorithms that iteratively learn from data, improving their performance autonomously without explicit programming. In healthcare, these technologies have revolutionized diagnostic accuracy, personalized treatment planning, and prognostication by harnessing large volumes of clinical and imaging data [5]. Osteoporosis is particularly amenable to AI solutions due to its reliance on quantitative imaging for diagnosis, the silent nature of the disease until fracture occurs, and the urgent need for efficient and large-scale risk stratification in aging populations.

This narrative review aims to summarize and critically evaluate current perspectives on the landscape of AI and ML applications in osteoporosis, focusing on their roles in enhancing diagnostic precision, fracture risk prediction, and personalized management strategies. We set out to explore how AI-driven tools can optimize bone mineral density assessment, integrate clinical risk factors with imaging biomarkers, and facilitate early identification of high-risk patients. Through this analysis, the review aims to evaluate the potential of AI and ML to transform osteoporosis care, while critically addressing existing gaps, methodological limitations, and future directions for research and clinical integration.

The epidemiology, pathogenesis, and clinical implications of osteoporosis profoundly impact global public health. It is estimated that one in three women and one in five men over the age of 50 will experience an osteoporosis-related fracture in their lifetime, with hip fractures associated with a significant reduction in survival [2,6,7]. The disease pathogenesis involves an imbalance in bone remodeling, driven by factors such as hormonal changes, genetics, and lifestyle [8,9]. Early diagnosis, primarily via DXA scanning and clinical risk assessment, is critical for initiating interventions that can reduce fracture risk [10,11,12]. Early management reduces fracture risk and preserves long-term function, while delayed diagnosis continues to raise morbidity, underscoring the need for stronger screening efforts [13]. The growing global burden demands early detection and targeted prevention strategies [14,15,16]. Emerging complex methods in AI and ML offer potential for improved risk prediction, pattern recognition, and diagnostic support [17,18,19,20]. This demonstrates the urgent need for innovative approaches such as AI to improve screening, risk prediction, and personalized management strategies.

AI and ML offer powerful tools for this task. For a clinical readership, it is crucial to understand that convolutional neural networks (CNNs) are the dominant model for analyzing medical images (e.g., X-rays, CT scans), while algorithms like random forests and gradient boosting are often applied to structured clinical data to predict fracture risk. The outputs of these models—such as probability scores, risk classifications, or segmentation maps—can be integrated into clinical workflows to support decision-making.

## 2. Materials and Methods

We conducted a narrative review to summarize and critically evaluate the literature on the applications of Artificial Intelligence (AI) and Machine Learning (ML) in the diagnosis and clinical management of osteoporosis. This approach allows for a broad exploration of concepts, themes, and methodological trends without the formal systematic processes of a meta-analysis. To identify relevant literature, a search in electronic databases (PubMed, Embase, and Scopus) was performed for articles published from inception to January 2025. The search strategy incorporated controlled vocabulary (e.g., MeSH terms) and free-text keywords related to osteoporosis and AI-based technologies. Combined search terms included “osteoporosis,” “artificial intelligence,” “machine learning,” “deep learning,” “fracture risk prediction,” and “decision support systems.” Boolean operators (AND/OR) were used to refine the queries. The search was limited to studies in English involving human subjects.

The scope of the review included studies discussing the use of AI or ML for imaging interpretation, diagnostic enhancement, fracture risk assessment, or personalized therapeutic approaches in osteoporosis. We excluded articles not focused on osteoporosis, non-English publications, and non-research reports (e.g., editorials without original data). Reference lists of pertinent studies were also screened to identify further relevant publications. The selection process was conducted by one reviewer with verification by a second; any disagreements were resolved through consensus. As this is a narrative review, a formal PRISMA flow diagram and risk-of-bias assessment were not performed. The aim was to select illustrative and impactful studies across key domains to build a comprehensive critical perspective. The literature search yielded a body of evidence from which key studies were selected to illustrate applications, challenges, and trends. Emphasis was placed on recent, high-impact studies and systematic reviews to inform a critical discussion.

## 3. Results

Applications of artificial intelligence (AI) and machine learning (ML) in the diagnosis of osteoporosis have shown promising advances, particularly in image recognition and decision support systems. AI techniques, especially deep learning models, have been employed to analyze radiological data such as X-rays and dual-energy X-ray absorptiometry (DXA) scans to improve diagnostic accuracy and efficiency.

### 3.1. Image Recognition and Radiological Data Analysis

Recent advances in artificial intelligence, particularly deep learning, have demonstrated substantial efficacy in the automated analysis of radiological images for osteoporosis diagnosis. Convolutional neural networks (CNNs) trained on various anatomical radiographs including hip, spine, chest, and dental panoramic images have shown high diagnostic performance, with reported area under the ROC curve (AUC) values ranging from 0.70 to 0.9987 in studies with sample sizes from hundreds to tens of thousands, using reference standards such as DXA, quantitative CT, or clinical diagnosis [21,22,23]. These AI models enable rapid and reliable estimation of bone mineral density (BMD) and classification of osteoporosis status, significantly reducing the labor-intensive process of manual image segmentation, traditionally undertaken by expert radiologists. For instance, automated AI systems such as “OsPor-screen” have been documented to be able to process chest radiographs in less than four seconds with accuracy comparable to that of human experts [21]. The integration of AI facilitates opportunistic screening by enabling retrospective analysis of routine radiographs without additional imaging or cost, expanding diagnostic reach, especially in resource-limited settings where dual-energy X-ray absorptiometry (DXA), the clinical gold standard, is less available. Nonetheless, performance variability persists due to confounding factors such as soft tissue artifacts, radiographic quality, and incidental findings, necessitating robust preprocessing and clinical data integration for optimized accuracy. It is important to note that many of these high AUC figures come from internal validation; robust external validation studies are less common. Taken together, AI-powered image recognition represents a promising direction toward scalable, efficient, and accessible osteoporosis diagnosis through widespread radiological datasets [21,22,23]. However, a critical evaluation reveals structural and methodological concerns in the evidence base, including spectrum bias, variability in validation rigor, and often under-reported performance in clinically relevant subpopulations (Table 1).

### 3.2. AI-Based Decision Support and Diagnosis

Artificial intelligence-based decision support systems (DSS) incorporate multidimensional clinical and imaging data to enhance early identification and risk stratification of patients predisposed to osteoporosis. By leveraging machine learning algorithms, these systems synthesize complex datasets comprising demographic variables, clinical risk factors, and imaging biomarkers to provide personalized fracture risk predictions and diagnostic classification [26]. It has been demonstrated that ML-fueled DSS can improve diagnostic reproducibility by minimizing interobserver variability and facilitating standardized clinical decision-making [27]. Models trained on large, heterogeneous populations aim for robustness in handling incomplete or variable-quality inputs, thereby supporting clinical workflows across diverse healthcare environments. Moreover, ensemble approaches integrating radiographic features with patient-specific covariates have yielded improved accuracy and predictive validity compared to image-only models [28]. Such systems not only assist clinicians in early diagnosis but also have the potential to guide therapeutic interventions and monitor treatment response, thus advancing precision medicine in osteoporosis management. AI and ML applications in imaging analysis and decision support are transforming osteoporosis diagnosis by increasing accuracy, enabling early detection, and expanding access to screening. Future research must focus on validating these tools in real-world clinical settings, standardizing methodologies, and addressing ethical and technical challenges for widespread adoption [29]. A critical perspective notes that many proposed systems remain in the developmental or early validation phase, with limited evidence of impact on final patient outcomes such as fracture reduction.

### 3.3. Fracture Risk Prediction

Predictive modeling of fracture risk in osteoporosis using artificial intelligence (AI) and machine learning (ML) has shown substantial promise in enhancing early identification and targeted intervention for high-risk patients. ML algorithms leverage diverse clinical and demographic data to build risk stratification models that often outperform traditional statistical approaches in discrimination metrics [30].

State-of-the-art ML models incorporate variables commonly associated with fracture risk, including age, body mass index (BMI), bone mineral density (BMD) T-scores, prior fracture history, history of falls, gender, chronic comorbidities, weight, height, and radiomics features extracted from imaging studies. A meta-analysis of 53 studies encompassing over 15 million patients suggested that ML predictive models achieve a pooled concordance index (C-index) of approximately 0.75 in both training and validation cohorts, indicating good discriminative ability for fracture risk prediction [31]. It is crucial to interpret this finding critically: while the pooled C-index suggests promising results, the analysis included heterogeneous models and populations, and the improvement over established tools like FRAX is often marginal in external validation. Furthermore, metrics of model calibration and clinical utility (e.g., net benefit) are frequently unreported. While ML models often show numerically higher C-indices than traditional tools such as FRAX in comparative studies, the extent of improvement varies, and ensemble methods such as gradient boosting often demonstrate high performance. Notably, in some studies (see Table 2) deep learning methods—particularly convolutional neural networks (CNNs)—have demonstrated superior performance compared to traditional algorithms, such as logistic regression and decision trees, with C-index values as high as 0.98 in validation sets [32]. Such high figures often stem from optimized internal validation and may not reflect generalizable performance.

These predictive frameworks are increasingly integrated with electronic health record (EHR) data, exploiting real-time clinical observations, laboratory results, and longitudinal health information to refine risk prediction and facilitate dynamic patient monitoring. For example, ML-based models developed from large-scale population health datasets enable community healthcare providers to screen individuals at elevated risk of osteoporosis and fractures through routine clinical variables, reducing reliance on resource-intensive imaging modalities such as dual-energy X-ray absorptiometry (DXA). This approach supports the implementation of personalized preventive strategies and judicious allocation of healthcare resources by prioritizing high-risk patients for further diagnostic assessment or therapeutic interventions [31]. Moreover, ML techniques such as ensemble learning, support vector machines (SVM), random forests, and artificial neural networks (ANN) have been successfully applied to predict not only overall fracture risk but also site-specific fracture occurrence (e.g., vertebral, hip fractures), enhancing clinical decision-making precision. Models combining clinical risk factors with radiomics data from imaging modalities further improve predictive accuracy by capturing subtle structural bone characteristics indicative of fragility [33].

Despite these advances, challenges remain in model generalizability due to heterogeneity of patient populations and limited external validation in many studies. Future directions emphasize rigorous prospective validation of ML fracture risk prediction models, integration with EHR systems for continuous learning and updating, and transparency in algorithm development to ensure clinical confidence and ethical use [34].

### 3.4. Personalized Treatment and Management

Personalized treatment approaches in osteoporosis management increasingly leverage artificial intelligence (AI) and machine learning (ML) by utilizing historical data on individual treatment responses to tailor therapeutic strategies. These AI systems analyze diverse datasets that encompass patient demographics, baseline bone mineral density (BMD), comorbid conditions, prior pharmacologic interventions, biochemical markers, and clinical outcomes to identify patterns associated with favorable or adverse responses to specific osteoporosis medications. Through such predictive modeling, machine learning algorithms aim to facilitate the optimization of treatment regimens by suggesting the most effective agents for individual patients, minimizing trial-and-error prescribing and enhancing therapeutic efficacy [35].

Retrospective analyses using ML frameworks enable stratification of patients based on their likelihood of response to antiresorptive agents (e.g., bisphosphonates, denosumab) or anabolic therapies (e.g., teriparatide, romosozumab). Algorithms such as random forests, gradient boosting machines, and artificial neural networks integrate multidimensional clinical data to predict fracture risk reduction and identify non-responders early. This approach supports clinicians in formulating personalized treatment plans that are dynamically adaptable, considering changes in patient status over time. For example, AI-driven insights may recommend modifications in dosing intervals, combination therapies, or prompt switching treatments in cases of suboptimal response or adverse events [36,37]. However, this field is largely exploratory, with very few validated models ready for clinical use. Evidence comes primarily from post hoc analyses of trial data, and lacks prospective validation.

In addition to predictive modeling of treatment outcomes, AI-enabled tools for monitoring are emerging. Wearable devices and mobile applications constitute potential tools for monitoring and managing osteoporosis therapies. These technologies could track real-time patient adherence, physical activity levels, fall risk, and other variables critical for effective osteoporosis management [38]. Wearables equipped with accelerometers and gyroscopes can detect gait instability and near-fall events, triggering alerts and enabling preventive interventions. Smartphone apps can facilitate patient engagement through medication reminders, educational content, and symptom reporting [39]. However, most of the reported applications in this domain are generic for chronic disease or fall prevention; osteoporosis-specific examples of AI-driven adherence monitoring or remote management are still largely anticipatory or in early development, with limited peer-reviewed evidence of effectiveness.

Combining AI with remote monitoring offers several potential advantages: improved adherence to pharmacotherapy, timely identification of complications or treatment failure, and enhanced patient-centered care through personalized feedback. Furthermore, continuous data capture enables longitudinal assessment of therapeutic efficacy in real-world settings, supplementing clinical trial evidence. This paradigm fosters precision medicine in osteoporosis by bridging the gap between static clinical snapshots and dynamic disease progression. Despite these promising developments, challenges such as data privacy, interoperability of devices with health records, and validation of predictive algorithms in diverse populations remain [40].

## 4. Discussion

The integration of artificial intelligence (AI) and machine learning (ML) into osteoporosis clinical practice offers several significant benefits along with important challenges related to ethics, legal frameworks, and implementation (Table 3).

AI and ML provide potential for enhanced diagnostic accuracy, sensitivity, and specificity for osteoporosis detection and classification beyond traditional methods such as dual-energy X-ray absorptiometry (DXA). Deep learning algorithms, particularly convolutional neural networks, can analyze medical imaging modalities like CT scans and radiographs rapidly and with precision comparable or superior to expert radiologists, allowing for opportunistic screening and earlier diagnosis especially in resource-limited settings where DXA is not widely available. Furthermore, AI systems efficiently handle and integrate multidimensional clinical data, including demographics, risk factors, and imaging biomarkers, facilitating personalized risk stratification and fracture risk prediction. These capabilities support improved clinical decision-making, reduce human subjectivity and interobserver variability, and enable dynamic monitoring of disease progression and therapeutic response. AI-driven models also aim to optimize resource allocation by targeting high-risk patients for timely intervention, potentially reducing fracture incidence and associated morbidity [30,41].

Despite their promise, AI and ML applications in osteoporosis face notable challenges. A critical appraisal of the evidence reveals frequent limitations, including spectrum bias in hospital-based cohorts, small sample sizes for some imaging models, and a lack of external validation across diverse populations, ethnic groups, and care settings (e.g., primary care vs. specialist clinics). Performance in specific subpopulations like men, the very elderly, or patients with secondary osteoporosis is often under-reported. Furthermore, while discrimination (e.g., AUC, C-index) is commonly reported, metrics of calibration and clinical usefulness, such as decision curve analysis, are less frequently addressed. Ethically, these systems require transparency and explainability to maintain clinician and patient trust, as “black-box” models can impede informed decision-making. Privacy concerns arise from handling sensitive patient data, necessitating compliance with data protection regulations. In osteoporosis, specific ethical issues relate to the opportunistic use of existing imaging (e.g., chest CT for lung disease) for AI-based screening, raising questions about consent for secondary data use, management of incidental findings, and potential overdiagnosis. Legally, the delegation of diagnostic and treatment recommendations to AI involves liability ambiguities in cases of error or harm, requiring clear regulatory frameworks and guidelines. The path to clinical adoption is also guided by evolving regulations for AI-based medical devices (Software as a Medical Device) in jurisdictions like the EU and USA, emphasizing the need for rigorous clinical evaluation and post-market surveillance. From an implementation perspective, many AI models suffer from limited external validation, lack of generalizability across diverse populations, and insufficient real-world clinical trials, which complicate widespread adoption. Integration into existing electronic health record (EHR) systems and clinical workflows demands interoperability standards and clinician training. Additionally, biases in training datasets may perpetuate health disparities if not properly addressed [41,42].

Robust validation through large-scale, multi-center prospective studies is essential to establish the clinical efficacy, reproducibility, and safety of AI-based osteoporosis tools. Standardization of model development, reporting, and evaluation metrics (such as AUC, sensitivity, specificity) is necessary to compare and benchmark algorithms accurately. Harmonizing imaging acquisition protocols and clinical data inputs will further enhance model reliability across healthcare settings. Moreover, ongoing updates and monitoring of AI systems in clinical use are crucial to maintain performance as underlying populations and technologies evolve. The development of specific regulatory guidelines for AI diagnostics and decision support in osteoporosis will facilitate responsible translation into clinical practice while safeguarding patient well-being [43].

**Table 3:** Key applications, benefits, and critical challenges of AI and ML in osteoporosis.

Application Area	Data Sources/Inputs	AI/ML Methods Used	Reported/Potential Benefits	Critical Challenges and Considerations	References
Diagnosis	Radiological images (X-rays, DXA, CT); Clinical risk factors	Convolutional Neural Networks (CNNs); Deep learning	Improved accuracy and speed of BMD assessment; Opportunistic screening.	Image quality variability; lack of standardization; limited generalizability; most evidence from internal validation; risk of spectrum bias.	[21,22,23,24,25]
Fracture Risk Prediction	Demographics; Clinical history; EHR data; Imaging radiomics	Random Forests; Gradient Boosting; Deep Neural Networks	Personalized risk stratification; Often improved discrimination vs. FRAX.	Heterogeneous data; limited external validation; Poor reporting of calibration and clinical utility; direct impact on fracture outcomes unproven.	[11,30,31,32,33]
Personalized Treatment	Patient therapy history; Biomarkers; Wearable data	Predictive analytics; Reinforcement learning	Optimized therapeutic selection; Early ID of non-responders.	Largely exploratory; few validated models; data privacy concerns; clinical efficacy unproven.	[35,36,37]
Decision Support Systems	Multimodal clinical data; Imaging biomarkers	Ensemble learning; Explainable AI	Reduced clinician workload; Standardized processes.	“Black-box” problem; Legal liability; need for clinician training and acceptance.	[26,27,41]
Monitoring and Management	Wearables (activity, falls); Patient-reported outcomes	Time-series models; Anomaly detection	Potential for real-time adherence and fall monitoring.	Osteoporosis-specific examples are limited; data accuracy and integration challenges; privacy.	[33,38,39]

## 5. Conclusions

This narrative review highlights the transformative potential of artificial intelligence (AI) and machine learning (ML) in the diagnosis, risk prediction, and personalized management of osteoporosis. AI-powered image recognition systems have shown promising accuracy in analyzing radiological data, enabling earlier and more accessible detection of osteoporosis beyond traditional methods such as dual-energy X-ray absorptiometry (DXA). ML models for fracture risk prediction show promise, often outperforming conventional tools in discrimination metrics, while AI-driven personalized treatment and monitoring approaches are emerging.

However, this review also identifies substantial limitations in the current evidence base. There is a notable lack of quantitative synthesis (e.g., meta-analysis) to meaningfully pool performance estimates. The robustness of many models is questionable due to insufficient external validation and testing across diverse clinical settings. Discussion of negative results or models that failed to outperform traditional methods is often missing, creating publication bias. Significant limitations of current AI evidence often include small sample sizes, retrospective design, heterogeneity in methodology, and a lack of evidence that these tools improve patient outcomes, such as fracture rates.

The importance of technological innovation in osteoporosis management cannot be overstated. AI and ML provide unparalleled opportunities to overcome limitations of current diagnostic and prognostic paradigms, optimize resource allocation, and enable precision medicine tailored to individual patient profiles. As the global burden of osteoporosis continues to rise with aging populations, these tools promise to enhance early detection, improve intervention timeliness, and ultimately reduce fracture incidence and associated morbidity. Successful integration of AI technologies into routine clinical workflows requires more than technical development; it necessitates rigorous research, transparent reporting of both positive and negative findings, addressing ethical and regulatory hurdles, and developing clear regulatory frameworks, all of which will be pivotal in realizing their full potential to revolutionize osteoporosis care and improve patient outcomes worldwide.

## Figures and Tables

**Table 1:** Selected studies on artificial intelligence (AI) and machine learning (ML) for osteoporosis imaging.

Modality	Study (Author, Year)	Sample Size (Train/Validate)	AI/ML Model	Key Performance (AUC)	Reference Standard	Validation Type and Notes
Various Radiographs	He et al., 2024 (Review) [22]	Literature review	Deep Learning Models (primarilsy CNNs)	AUC range 0.70–0.9987	Various	Review summarizing wide performance ranges; underscores need for standardized reporting.
Dental Panoramic X-ray	Sukegawa et al., 2022 [23]	1179 patients	Ensemble Deep Learning (CNN + LightGBM)	AUC: 0.891 (with clinical data)	DXA	Single-center study; internal validation. Model integrates radiomic features and clinical covariates (age, gender).
Computed Tomography (CT)	Ong et al., 2023 (Review) [24]	Synthesis of multiple studies	Various CNNs and traditional ML	High accuracy across studies (AUC 0.70–0.99+)	DXA, QCT, Biomechanics	Systematic review; notes CT-based AI can assess BMD and bone microstructure but highlights heterogeneity in methods.
Chest X-ray (CXR)	Que et al., 2025 [25]	4106 patients (3047 train/1059 test)	Commercial AI Software (DL-based)	AUC: 0.92 for osteoporosis detection	DXA	External validation cohort; demonstrates potential for opportunistic screening on routine chest CTs.

**Table 2:** AI/ML for osteoporosis fracture risk prediction—key studies.

Study/Model (Author, Year)	Cohort and Sample Size	Predictors Used	ML Model (s)	Performance (C-Index/AUC)	Comparison to Traditional Model	Key Finding/Note
Hong et al., 2024 [30]	Perspective/Review	Clinical data, EHRs, imaging biomarkers	Not specified (general AI/ML)	Discusses improved accuracy over conventional methods	Positions AI as a tool to enhance, not replace, FRAX	Highlights need for large-scale trials to prove AI’s impact on fracture outcomes.
Tu et al., 2024 [32]	Nationwide cohort (Taiwan), N = 37,955	46 clinical variables from health check-ups (no BMD)	Extreme Gradient Boosting (XGBoost)	AUC: 0.861	Outperformed logistic regression (AUC: 0.781)	Demonstrates effective risk prediction using clinical data without imaging; requires external validation.
Wu et al., 2023 (Meta-analysis) [31]	53 studies (>15 million patients)	Clinical variables, BMD, imaging radiomics	Various (RF, GB, SVM, ANN, DL)	Pooled C-index: 0.75 (95% CI: 0.72–0.78)	ML models generally showed higher discrimination than traditional statistical models	Broad evidence synthesis; highlights ML’s predictive potential but notes significant heterogeneity, risk of bias in included studies, and lack of quantitative synthesis on clinical impact.
Ulivieri et al., 2025 (Review) [33]	Review of current evidence	Multimodal (clinical, BMD, imaging, biochemical)	AI/ML frameworks	Summarizes state-of-the-art performance, often exceeding FRAX in discrimination	Discusses integration of AI to augment FRAX	Focuses on clinical implementation pathways and remaining challenges (validation, regulation).

## Data Availability

No new data were created or analyzed in this study.
